# *Morchella* Polysaccharides in Oxidative Stress—Structure–Activity Relationships, Redox Signaling, and Translational Evidence

**DOI:** 10.3390/nu18182981

**Published:** 2026-09-11

**Authors:** Na Xu, Yi Lu

**Affiliations:** 1 Department of Translational Genomics, Faculty of Health, Medicine & Life Sciences, GROW Institute for Oncology and Reproduction, Maastricht University, 6200 MD Maastricht, The Netherlands; 2 Department of Human Genetics, Radboud University Medical Center, 6525 GA Nijmegen, The Netherlands; luyi920127@163.com

**Keywords:** *Morchella*, polysaccharides, oxidative stress, redox signaling, Nrf2, mitochondria, structure–activity relationship, gut microbiota

## Abstract

*Morchella* polysaccharides are commonly labeled as antioxidants because they quench radicals in chemical assays, yet this designation does not establish biological redox efficacy. This critical narrative review reframes these fungal polymers as candidate redox-modulating biomacromolecules. A structured search of PubMed and OpenAlex through 17 August 2026 yielded 84 unique records after deduplication; 31 core *Morchella*-polysaccharide reports with redox-relevant experimental evidence were mapped, supplemented by five mechanistically adjacent studies. Evidence was graded from chemical assays (E1) to human intervention (E5), with a separate causality score (M0–M2). Structure-resolved studies indicate that molecular weight, branching, charge, conformation, source, and acetylation or degradation can modify activity, but process-related co-variation and incomplete purity controls preclude universal structure–activity rules. Cellular and animal studies repeatedly implicate PI3K/Akt–Nrf2/HO-1, AMPK/Sirt1, mitochondrial apoptosis, NF-κB/NLRP3, and gut-mediated pathways, although most reported links remain associative. Within the databases and search strategy used, no eligible human intervention study was identified. We propose a translational roadmap requiring identity-controlled materials, orthogonal redox endpoints, causal pathway perturbation, exposure and microbiota studies, and standardized early-phase trials. The field should move beyond DPPH-positive claims toward reproducible mechanisms and biologically credible redox protection.

## 1. Introduction

Oxidative stress is not synonymous with the mere presence of reactive oxygen species (ROS). It is a disturbance of redox homeostasis in which oxidant production, antioxidant buffering and redox signaling become mismatched, leading to inappropriate molecular modification and loss of adaptive control [1,2,3]. This distinction matters for food-derived macromolecules. A compound can reduce a stable chromogenic radical in a tube yet fail to reach a biological target, while a molecule with modest electron-donating chemistry can protect cells by inducing endogenous defenses, changing mitochondrial metabolism or reshaping gut microbial metabolites.

Oxidative stress and inflammation are tightly linked: excess ROS can activate inflammatory signaling and inflammasome pathways, whereas cytokine production, immune-cell recruitment, and inducible oxidant-generating enzymes can further intensify oxidative stress [4,5]. This bidirectional amplification is relevant to the inflammatory and tissue-injury models reviewed below and is examined mechanistically in Section 5.5.

Most antioxidant reports on *Morchella* (morel) polysaccharides have emphasized DPPH, ABTS, hydroxyl-radical or superoxide scavenging, reducing power, and metal chelation. These assays are useful for screening and standardization, but they differ in solvent, radical source, stoichiometry, and kinetics; none alone demonstrates protection in a living system or identifies the constituent responsible for the apparent activity [6,7]. Malondialdehyde (MDA), total antioxidant capacity, and antioxidant-enzyme activities are likewise informative but can be method-sensitive and should be interpreted alongside direct injury and functional endpoints [8].

The evidence base has nevertheless changed. Defined fractions now protect oxidant-challenged cells and simple organisms. FMP-1 has been linked to PI3K/Akt, nuclear Nrf2, and HO-1 in H_2_O_2_-injured A549 cells, whereas mycelial MMP-L has been associated with PI3K/Akt and mitochondrial apoptosis in PC12 cells. Animal studies increasingly connect redox recovery with AMPK/Sirt1, NLRP3-related inflammation, and microbiota-associated metabolism [9,10,11,12]. Together, these observations motivate a transition from ‘antioxidant activity’ to oxidative-stress biology.

**Figure 1:**
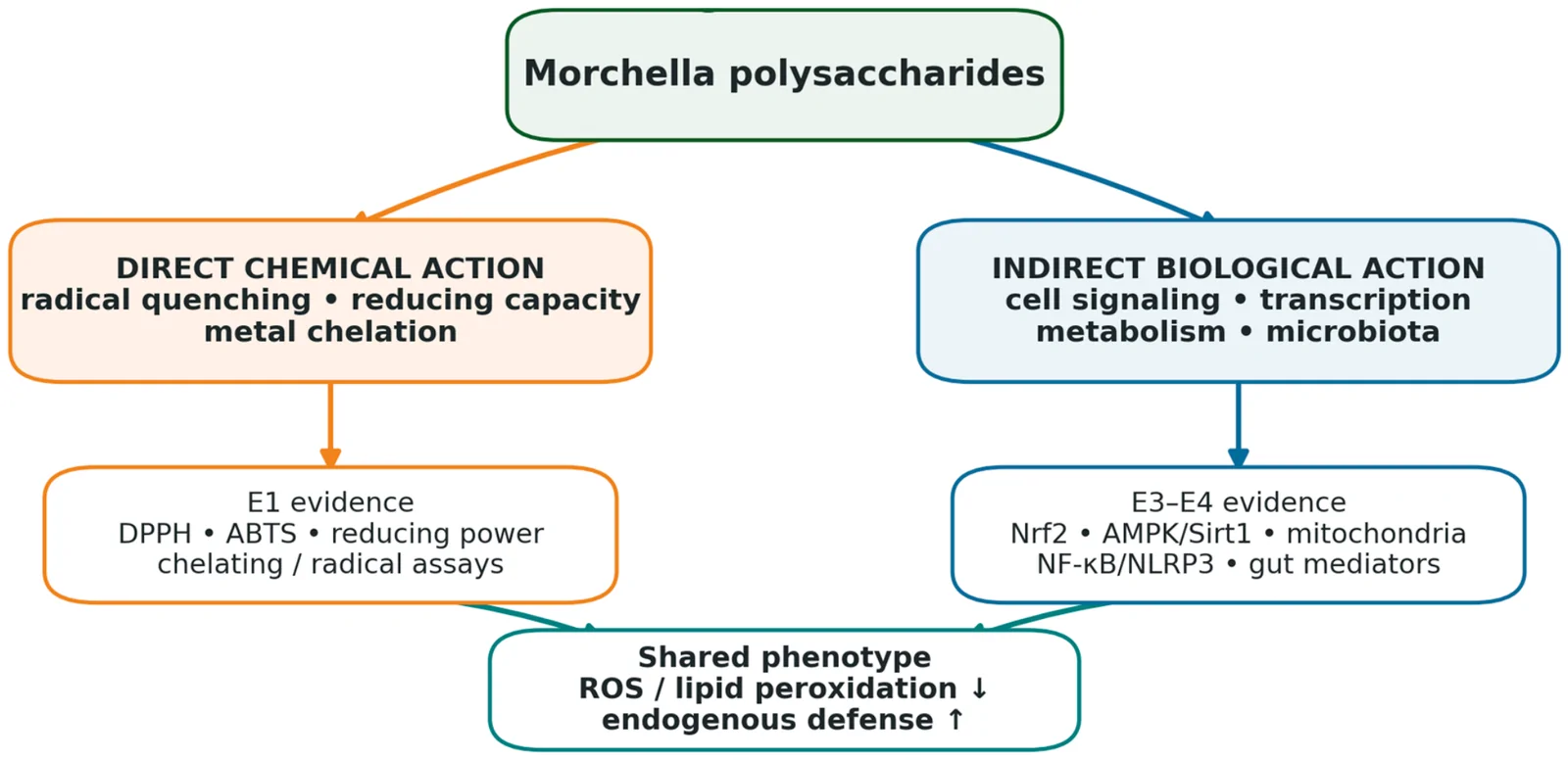
Direct versus indirect antioxidant action. E1 chemical reactivity can support quality control, whereas biological protection may arise from signaling, metabolic adaptation, mitochondrial preservation or microbiota-derived mediators. The two modes are not mutually exclusive, but they require different experiments.

A 2024 review comprehensively summarized the extraction, structure, and chemical antioxidant activity of *Morchella* polysaccharides [13]. The present review addresses a different question: How credible is the claim that these polymers regulate biological oxidative stress, which structural features might matter, and what evidence is required for translation? Its added value is fourfold: we (i) separate direct assay reactivity from biological redox modulation; (ii) grade biological relevance and mechanistic causality on independent axes; (iii) evaluate signaling, mitochondrial, inflammatory, and gut-mediated evidence against material identity and exposure; and (iv) propose sequential translational go/no-go criteria. In doing so, the review addresses persistent gaps in purity control, orthogonal redox measurement, causal pathway testing, exposure, microbiota mediation, and human evidence. The central proposition is that *Morchella* polysaccharides should increasingly be viewed as redox-modulating biomacromolecules rather than simply radical-scavenging antioxidants. The distinction between direct chemical reactivity and indirect biological redox modulation is summarized in Figure 1.

## 2. Literature Search Strategy and Evidence Appraisal

### 2.1. Search Scope and Record Identification

This article is a critical narrative review with a structured evidence map, not a registered systematic review or meta-analysis. Searches were completed on 17 August 2026. PubMed was queried from database inception using the following expression:

((Morchella[Title/Abstract] OR morel*[Title/Abstract]) AND (polysaccharide*[Title/Abstract] OR glucan*[Title/Abstract] OR exopolysaccharide*[Title/Abstract]) AND (oxidative[Title/Abstract] OR antioxidant*[Title/Abstract] OR ‘reactive oxygen species’[Title/Abstract] OR ROS[Title/Abstract] OR redox[Title/Abstract] OR ‘lipid peroxidation’[Title/Abstract] OR malondialdehyde[Title/Abstract] OR Nrf2[Title/Abstract] OR SOD[Title/Abstract] OR glutathione[Title/Abstract]))

The date limit was 1 January 1900 to 17 August 2026.

The PubMed search returned 50 records. To broaden coverage of food-science, carbohydrate and regional journals, OpenAlex was searched in title and abstract fields with ‘Morchella polysaccharide antioxidant’ (59 records), ‘Morchella polysaccharide oxidative stress’ (22) and ‘morel polysaccharide antioxidant’ (15); 67 OpenAlex records were unique. DOI- and title-based deduplication across sources produced 84 unique records for screening. Backward and forward citation chasing of the 2024 topic review and key mechanistic papers identified older or newly indexed studies that used nonstandard redox terminology. Thirty-one primary reports directly meeting the core redox criteria were mapped; five mechanistically adjacent SAR or mediator studies were retained to define hypotheses and experimental priorities.

### 2.2. Eligibility and Data Extraction

Studies were included when they (i) investigated a polysaccharide, defined carbohydrate-rich fraction, or chemically modified derivative derived from a *Morchella* fruiting body, mycelium, or culture broth and (ii) reported a chemical redox assay, biological oxidation system, oxidative-stress model, redox biomarker or pathway, or a directly compared structural modification. Primary research in English or Chinese was considered when methods and outcomes were interpretable. Publications in languages other than English or Chinese were excluded because their full methods and outcomes could not be evaluated reliably. Reviews were used for citation discovery and context, not as primary evidence.

We excluded studies of whole-mushroom extracts, isolated phenolics, peptides, or other non-polysaccharide constituents; studies of cultivation stress experienced by the fungus itself; and nanocomposites or delivery systems in which the polysaccharide contribution could not be separated. We also excluded non-redox bioactivity reports without a mechanistic bridge and records lacking primary experimental data. For each eligible study, we extracted source and species, product identity and purity, molecular-weight information, monosaccharides, linkages or conformation where available, model and stressor, dose, redox and injury outcomes, proposed pathway, causal perturbation, functional outcome, and major design limitations.

Because terminology and assay conditions were heterogeneous, no quantitative pooled effect was attempted. Counts reflect the structured search and evidence map rather than a claim of exhaustive global retrieval. The evidence map retains the documented search cutoff of 17 August 2026 so that the reported record counts and screening decisions remain auditable; accordingly, claims of absence are explicitly bounded by the databases and search strategy used.

### 2.3. Two-Dimensional Evidence Grading

Biological relevance and mechanistic causality were deliberately scored on separate axes. E1 denotes chemical radical, reducing or chelation assays, E2 denotes protection of a biological substrate in a cell-free oxidation system, E3 denotes cellular or simple-organism oxidative challenge, E4 denotes a mammalian model with tissue-level redox outcomes, and E5 denotes human intervention. At any E level, M0 indicates biomarker or pathway association; M1 indicates a pharmacological inhibitor/agonist or rescue test; and M2 indicates genetic loss/gain, direct target engagement, or mediator depletion/transfer. Thus, an E4/M0 mouse study may be biologically closer to humans than an E3/M1 experiment while still offering weaker causal pathway evidence.

**Figure 2:**
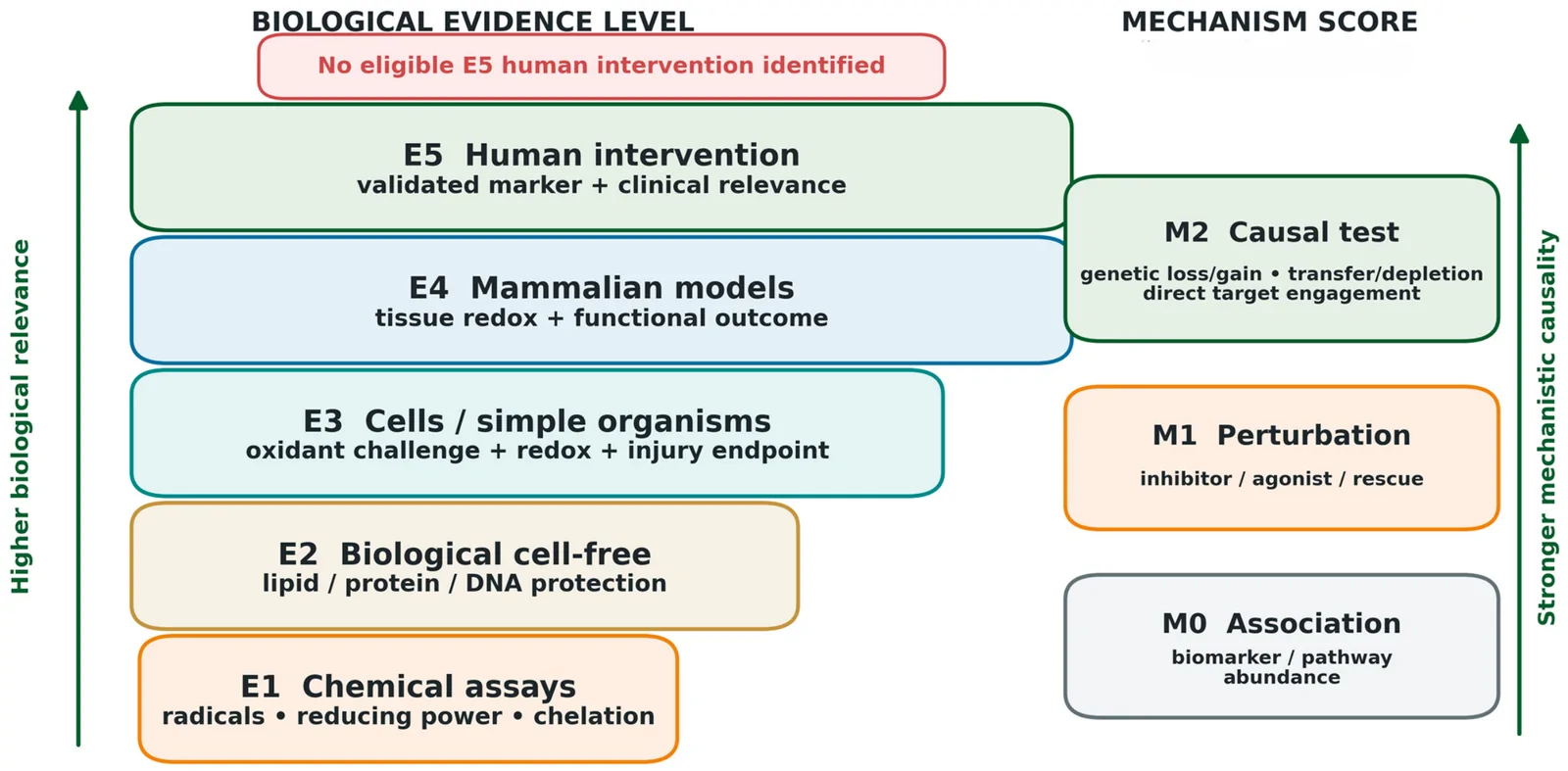
Evidence hierarchy for redox claims and an independent causality overlay. E1–E5 rank biological context; M0–M2 rank the strength of mechanism testing. Within the databases and search strategy used, no eligible E5 human intervention study was identified through 17 August 2026.

This framework separates biological context from causal inference: DPPH positivity does not establish in vivo efficacy, and changes in Nrf2 or the microbiota indicate association unless necessity or mediation is tested. A rare M1 example is the macrophage study in which sodium protoporphyrin IX partially reversed the HO-1-dependent suppression of NO/iNOS while leaving the NF-κB effect largely distinct [14]. An adjacent M2 example is a 2026 myocardial-inflammation study in which antibiotics abolished the protective effect of a *Morchella* polysaccharide, providing causal support for a gut-microbial mediator even though oxidative stress was not its primary endpoint [15]. The two-dimensional grading framework is summarized in Figure 2.

## 3. Chemical and Structural Diversity Relevant to Redox Biology

### 3.1. Biological Source Is Part of Material Identity

‘*Morchella* polysaccharide’ is not a single substance. Fruiting bodies, stipes, submerged mycelia and extracellular culture products differ in biosynthesis, developmental stage, medium exposure and coextractives. Even within one species, hot-water, enzyme-assisted, deep-eutectic-solvent and subcritical-water extraction generate different yields, molecular-weight distributions and physicochemical profiles [16,17,18,19,20,21]. A biologically meaningful identity therefore requires, at minimum, taxonomically authenticated source material, tissue/culture origin, batch, extraction and purification history, carbohydrate and protein content, endotoxin testing and a molecular-weight distribution.

Source comparisons can be informative when the analytical and biological platforms are shared. For example, fruiting-body MSF from *M. esculenta* was glucose-rich, whereas MSL isolated from the *M. esculenta* fermentation broth was mannan-rich and contained trace glucuronic acid; the two preparations differed in molecular weight and produced different transcriptomic and microbiota-associated responses in a zebrafish model [22]. However, biological source, composition, and production process changed together. The study generates hypotheses but cannot assign the phenotype to mannose, molecular weight, or a specific linkage.

### 3.2. Size, Linkage, Branching, Charge and Conformation

Reported *Morchella* fractions span low-kilodalton glucans to multi-megadalton heteropolysaccharides. FMP-1 (approximately 4.7 kDa) is a glucose-rich fraction containing mannose and galactose and has been studied across chemical, zebrafish and cell models [9,23]. At the other extreme, MMP-L (3.02 MDa) and a 2.283 MDa MSP remain active in cellular or animal models [10,24]. These observations immediately reject a universal linear rule that lower molecular weight necessarily means greater biological efficacy.

Monosaccharide composition is an incomplete structural description. Linkage position, anomeric configuration, branch density and higher-order conformation determine epitope display, solubility, viscosity and enzyme accessibility. In *M. sextelata*, MSP1-1 was described as a branched (1 → 4)-α-D-glucan with random-coil behavior, whereas MSP1-2 contained a galactomannan/α-glucan network and was much smaller [25]. Both showed E1 activity, but their biological disposition was not compared. This is precisely where the field needs structure-matched cellular and receptor experiments rather than broader chemical-assay panels.

### 3.3. Modification as a Quasi-Experimental SAR Tool

Chemical and physical modifications can create within-parent comparisons that are more informative than across-study correlations. Sulfation, carboxymethylation and acetylation of a parent *M. esculenta* polysaccharide changed E1 activity, with the acetylated derivative (degree of substitution of 0.40 ± 0.07) performing best in that series [26]. Gamma irradiation lowered apparent molecular weight and improved chemical and PC12-cell protection [27]. More recently, snailase degradation followed by acetylation produced EMP and AEMP from a common parent; AEMP gave the strongest chemical antioxidant and DSS-colitis effects [28].

Even these designs do not isolate one determinant automatically. Degradation changes reducing-end abundance and polydispersity; sulfation changes ionic strength and metal chelation; and acetylation changes solubility, hydrophobicity and fermentability. Modifications may also alter endotoxin binding or residual small-molecule retention. A rigorous modification study must therefore report substitution position and degree, molar mass by SEC-MALS where feasible, solution conformation in the assay medium, mass balance, and matched purity and exposure. Representative structure-resolved and structure-modified preparations are summarized in Table 1.

### 3.4. Functional Groups and Mechanistic Implications

No single ‘antioxidant group’ has been established for *Morchella* polysaccharides. Accessible reducing ends and hydroxyl-rich microenvironments may support electron or hydrogen transfer in E1 assays, whereas uronic acid, sulfate, and carboxymethyl groups can increase anionic charge, metal coordination, and hydration. Acetyl groups can alter electron distribution, hydrophobicity, solubility, and chain conformation rather than acting as a simple antioxidant moiety [20,26,27,28]. These properties can influence both assay chemistry and the exposure of a preparation to cells, proteins, or microbes.

Glycosidic linkage, branching, and higher-order conformation are not redox groups themselves, but they can control epitope display, multivalent receptor sensing, viscosity, aggregation, and digestive or microbial accessibility [25]. A structural feature may therefore affect biological redox outcomes indirectly through receptor- or gut-mediated signaling rather than through direct radical quenching. Because current modification studies usually change several variables simultaneously, these mechanisms remain testable hypotheses rather than established structure–activity rules.

## 4. From Radical Scavenging to Biological Oxidative-Stress Protection

### 4.1. What Chemical Assays Can—and Cannot—Establish

E1 assays remain useful for rapid fractionation, lot comparison and mapping how processing changes reducing capacity or chelation. Early extracellular-polysaccharide work established dose-dependent scavenging and reducing activity, and enzyme-assisted or modified preparations often outperformed their parent or conventionally extracted material [16,18,19,26]. FMP-1, for example, showed reported half-maximal values of approximately 74.3 μg/mL for hydroxyl radicals, 119.3 μg/mL for DPPH and 161.5 μg/mL for superoxide in the original assay systems [23].

These numerical values are not portable potency constants. DPPH favors reactions in organic or mixed solvents, whereas ABTS is accessible to both hydrophilic and lipophilic compounds. Hydroxyl-radical assays often depend on metal-driven chemistry, and reducing-power readouts can be dominated by reducing ends or residual phenolics. DPPH is therefore a preliminary screen and cannot identify the constituent responsible for the signal. Color, turbidity, viscosity, and light scattering from polysaccharide preparations can further distort absorbance. Activity attribution should combine blank-corrected kinetics and at least two chemically orthogonal assays with carbohydrate, protein, and phenolic measurements; LC–MS fingerprinting of low-molecular-weight coextractives; and, where feasible, activity-guided depletion and reconstitution [6,7]. Concentrations should be reported on both product-mass and carbohydrate-mass bases.

Cell-free protection of a biological substrate (E2)—for example, suppression of lipid, protein or DNA oxidation under a defined radical flux—would be more informative than another chromogenic-radical result. Surprisingly few *Morchella* studies occupy this intermediate tier. Filling E2 would help distinguish generic reducing activity from protection of vulnerable biomolecular targets before moving into complex cells.

### 4.2. Cellular and Simple-Organism Models

H_2_O_2_-challenged PC12, A549, HEK293T and H9C2 cells are the dominant E3 systems. Across these models, *Morchella* polysaccharides generally reduce probe-detected ROS, MDA, lactate dehydrogenase leakage, nuclear condensation or apoptosis while increasing SOD, CAT, GSH-Px, GSH or total antioxidant capacity [9,10,29,31,32]. The convergence is encouraging because injury and redox endpoints move together. However, H_2_O_2_ dose and exposure time vary widely, ROS probes are susceptible to artifacts, and milligram-per-milliliter polysaccharide concentrations can alter viscosity, osmolarity or probe access.

Zebrafish embryos and *Caenorhabditis elegans* offer whole-organism context without resolving mammalian oral exposure. FMP-1 reduced AAPH-induced ROS, nitric oxide, lipid peroxidation and embryo cell death while restoring antioxidant enzymes [23]. Cap- and stipe-derived polysaccharides improved H_2_O_2_ survival in *C. elegans* with lower ROS/MDA and increased gst-4, sod-3 and gsh-px expression [33]. These are valuable bridges between E1 and E4, especially when genetics can be used; future nematode studies should use RNA interference or mutants to test whether SKN-1/Nrf-like pathways are required.

### 4.3. Mammalian Models and the Absent Human Tier

E4 evidence spans D-galactose aging, CCl_4_ or alcohol liver injury, doxorubicin cardiotoxicity, DSS colitis, LPS intestinal injury, NAFLD and exercise fatigue. Common patterns are lower MDA or protein carbonyls, higher SOD/CAT/GSH-Px/GSH, reduced inflammatory cytokines, and improved tissue or performance outcomes [11,28,32,34,35,36,37]. These studies show that redox biomarkers can change in vivo; they do not yet prove that redox regulation is the initiating mechanism rather than a consequence of reduced injury, inflammation or lipid burden.

Within the databases and search strategy used, no eligible human intervention study testing a structurally characterized *Morchella* polysaccharide with predefined oxidative-stress endpoints was identified. This is the central translational gap. Animal doses are typically reported as milligrams per kilogram of a product with variable purity, and polymer absorption is rarely measured. Human work should not begin by repeating total antioxidant capacity alone; it should use a food-grade, batch-specified preparation, validated systemic markers such as F_2_-isoprostanes where appropriate, exposure or fermentation markers, and a functional endpoint tied to the intended population.

## 5. Molecular Mechanisms of Oxidative-Stress Protection

### 5.1. Direct Chemistry Versus Proximal Biological Sensing

A polysaccharide can contribute direct chemistry through reducing ends, hydroxyl-group environments, charged substituents, metal chelation and associated small molecules, yet most high-molecular-weight fungal polysaccharides do not resemble classical small-molecule reductants in diffusibility or redox stoichiometry. Their biological effects may begin, instead, at the cell surface, in endosomes, in the intestinal lumen or after microbial fermentation. Pattern-recognition receptors such as Dectin-1 can sense multivalent fungal β-glucan structures and reorganize cellular signaling, illustrating a plausible receptor-level paradigm even though direct receptor engagement has not been established for the principal *Morchella* redox fractions [38,39].

The field should therefore distinguish chemical availability (Can the material donate electrons or chelate metal under the assay conditions?) from biological availability (Does the intact polymer, a fragment or a microbial metabolite contact a relevant target?). Neither is implied by product mass alone. Receptor-binding, uptake, endosomal localization, digestion and fermentation experiments are prerequisites for connecting structure to a proximal mechanism.

### 5.2. PI3K/Akt–Nrf2/HO-1: A Leading but Incompletely Validated Axis

Nrf2 coordinates inducible antioxidant and detoxification programs. Under basal conditions, Keap1 promotes Nrf2 turnover; electrophilic, oxidative and signaling inputs stabilize Nrf2, promote nuclear accumulation and induce targets including HO-1 and NQO1 [40,41]. PI3K/Akt can intersect with this system through multiple context-dependent routes, but increased p-Akt, nuclear Nrf2 and HO-1 after treatment do not prove a linear PI3K → Akt → Nrf2 chain.

FMP-1 reduced H_2_O_2_-induced ROS, MDA, mitochondrial cytochrome-c release and caspase-3 activation in A549 cells while increasing SOD, total antioxidant capacity, Akt phosphorylation, nuclear Nrf2 and HO-1 [9]. This is a coherent M0 mechanism package. The decisive experiment would combine PI3K/Akt inhibition with Nrf2 knockdown or knockout, confirm loss of HO-1/NQO1 induction and determine whether redox and survival rescue disappear. The LPS-macrophage study provides a useful precedent: pharmacological HO-1 inhibition partly reversed NO/iNOS suppression, moving one branch from association toward M1 [14].

In zebrafish, changes in Keap1 and Nrf2 transcripts after treatment with *M. esculenta* fruiting-body MSF or fermentation-broth MSL were interpreted within the same broad pathway [22]. Transcript abundance, however, is not equivalent to Nrf2 stabilization, nuclear binding, or target-gene flux. Future studies should quantify nuclear Nrf2, ARE occupancy or reporter activity, and canonical targets, then test requirement genetically.

### 5.3. Mitochondrial Redox Control and Apoptosis

Mitochondria both respond to and amplify oxidative injury. Loss of membrane potential, permeability transition and outer-membrane permeabilization release cytochrome c, enabling caspase activation; the balance between anti-apoptotic Bcl-2 and pro-apoptotic BAX is a common regulatory readout [42]. NMCP-2 preserved mitochondrial membrane potential and ultrastructural integrity in H_2_O_2_-challenged HEK293T cells while increasing Bcl-2 and reducing BAX and cleaved caspase-3 [29]. Similar Bcl-2/BAX/caspase-3 patterns occur with FMP-1, stipe-derived MP-C, MMP-L and NMCP-2 in doxorubicin injury [9,10,32,43].

These convergent endpoints support mitochondrial preservation as a recurring phenotype. They do not establish whether mitochondria are the primary target. Lower mitochondrial injury can follow reduced extracellular oxidant load, Nrf2 activation, anti-inflammatory signaling or altered substrate metabolism. Higher-resolution work should measure compartment-specific ROS, oxygen consumption, membrane potential with orthogonal probes, permeability transition, cytochrome-c redistribution and mitochondrial morphology, combined with BAX/BAK loss-of-function or caspase rescue where appropriate.

### 5.4. AMPK/Sirt1 and Metabolic Redox Adaptation

AMPK responds to cellular energy stress and can coordinate catabolic flux, mitochondrial quality control and antioxidant adaptation. Sirt1 couples NAD^+^ availability to deacetylation programs; reciprocal AMPK–Sirt1 interactions link energy state to mitochondrial and redox homeostasis [44]. In HFD- and MCD-based NAFLD models, a *Morchella* polysaccharide improved lipid accumulation, inflammation and fibrosis with AMPK/Sirt1 signaling changes [11].

The NAFLD result is mechanistically relevant but should not be over-read as direct antioxidant action. Lower lipid burden reduces substrate for peroxidation, and improved insulin sensitivity can normalize mitochondrial electron pressure. AMPK or Sirt1 inhibition, time-resolved sampling and direct oxidative-damage measures are needed to determine whether redox improvement is upstream, downstream or parallel to metabolic correction.

### 5.5. NF-κB, NLRP3 and Redox–Inflammation Crosstalk

Oxidative stress and inflammation form a bidirectional network. ROS can affect NF–κB signaling and inflammasome activation, while cytokines, infiltrating leukocytes and inducible enzymes increase oxidant production [4,5]. *Morchella* polysaccharides frequently lower ROS/MDA and inflammatory mediators together. In H_2_O_2_-injured PC12 cells, MIP suppressed NF-κB and p38/JNK signals and increased ERK activity [31]. Sulfated FMP-1 derivatives reduced PM_2.5_-triggered ROS, apoptosis, TNF-α, IL-1β, iNOS and COX-2 with less NF-κB nuclear translocation [45].

Recent liver work connects recovered SOD/CAT/GSH and reduced MDA with lower expression of NLRP3/Ifi16/Pycard/pro-caspase-1-associated pyroptotic programs [12]. An Alzheimer-model study extended this pattern to NLRP3 pyroptosis, caspase-dependent apoptosis and RIPK1-associated necroptosis (PANoptosis) [24]. These data broaden the biological phenotype but remain M0. Genetic inflammasome or NF-κB perturbation, temporal ordering and redox-insensitive pathway controls are necessary to identify whether redox regulation drives the inflammatory change or simply accompanies less tissue damage.

### 5.6. Gut Microbiota as an Indirect Redox Mediator

Large dietary polysaccharides are often more likely to encounter the gut microbiota than peripheral cells. Fermentation can alter short-chain fatty acids, bile acids, indoles and lipid mediators; barrier effects can reduce systemic inflammatory oxidant production [46]. MEP2 in chronic alcohol injury, MEP in LPS intestinal injury, a *Morchella* exopolysaccharide in diabetes, and anti-fatigue or enzymatically degraded preparations have all been associated with microbiome and metabolome changes [30,36,37,47,48].

**Figure 3:**
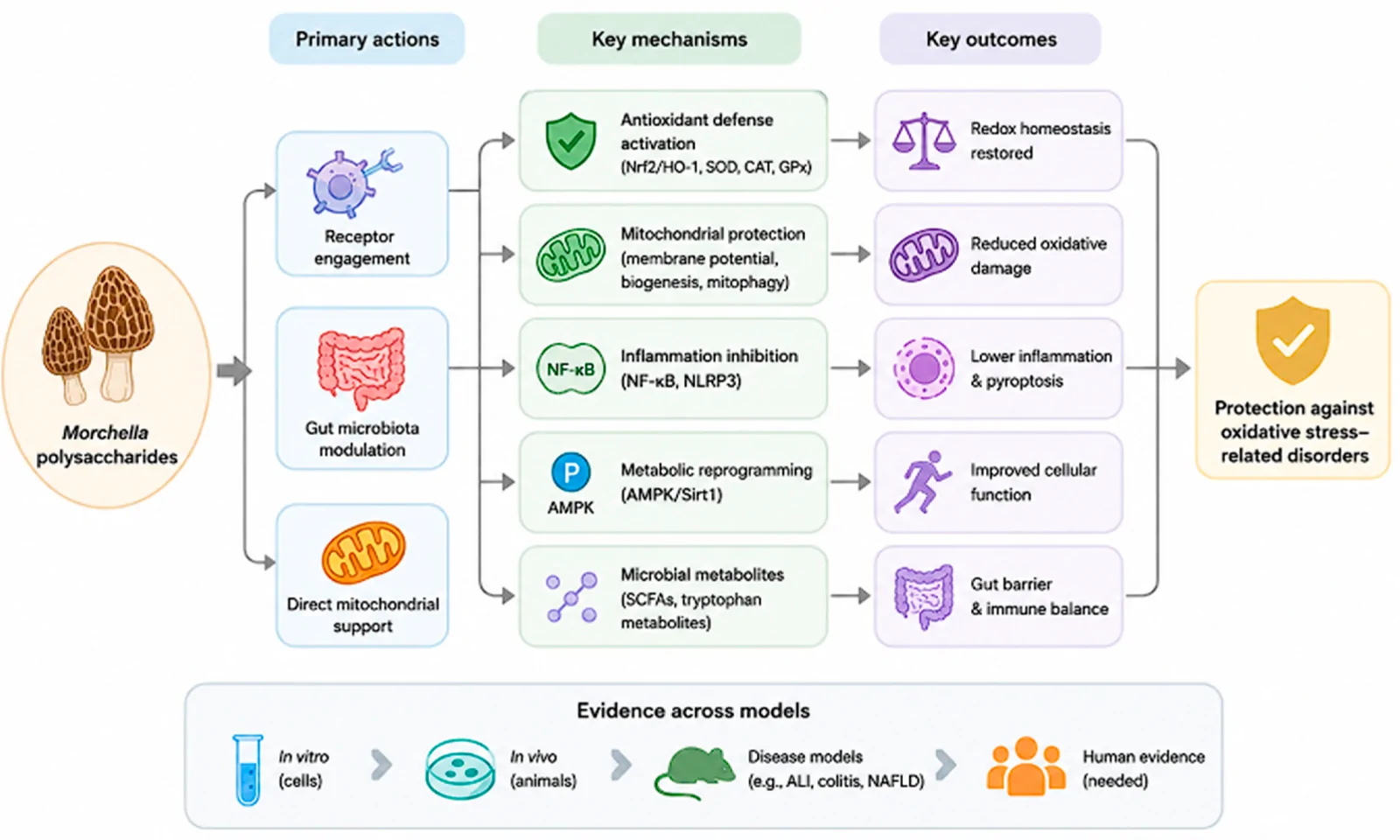
Illustrative working model of oxidative-stress protection by *Morchella* polysaccharides. The diagram integrates recurrent associations involving potential receptor or cell-surface sensing, gut microbiota modulation, mitochondrial preservation, antioxidant defense, inflammatory signaling, and metabolic adaptation.

Correlation is not mediation. The most instructive adjacent study used an antibiotic cocktail to abolish a myocardial protective effect and linked the phenotype to *Lactobacillus* and 12-HEPE, meeting an M2 mediator criterion [15]. Redox-focused studies should adopt the same logic: antibiotic depletion with careful controls, fecal transfer, gnotobiotic colonization, or direct metabolite rescue. Digestion and fermentation studies should connect structural disappearance of the parent polymer to the appearance of candidate active products and tissue-level redox outcomes. Figure 3 illustrates this working model; its arrows denote proposed or associative relationships. Representative studies across evidence tiers and their principal limitations are compared in Table 2.

## 6. Structure–Activity Relationships: What Is Supported and What Remains Hypothetical

### 6.1. Molecular Weight Is Likely Non-Monotonic

Lower molecular weight can increase aqueous solubility, reduce viscosity, expose reducing ends and improve diffusion through mucus or extracellular matrices. This logic is consistent with stronger E1/E3 activity after gamma irradiation, enzymatic degradation or subcritical-water extraction [21,27,28]. It is not a universal biological law. Multi-megadalton MMP-L protected PC12 cells, and high-molecular-weight MEP2 and MSP were active in animal models [10,24,30]. Large polymers may achieve multivalent receptor clustering, prolonged gut retention or superior fermentability. The likely relationship is context-specific and non-monotonic.

### 6.2. Composition, Linkages and Conformation Require Within-Parent Designs

Glucose-rich α-glucans dominate several purified fractions, but galactose, mannose, glucosamine and uronic acid occur in variable proportions. Cross-study correlations between a monosaccharide percentage and activity are especially weak because species, source, process, purity and assay all change simultaneously. Linkage and conformation are biologically more plausible determinants: branching controls multivalency and hydrodynamic size, whereas helices, coils and networks change epitope accessibility and aggregation. However, only a small subset of studies characterize these features, and even fewer test them on the same biological platform [25,49].

A decisive SAR program would start from one structurally resolved parent polymer. Controlled size reduction, debranching and substitution would generate a factorial series; each product would be matched for carbohydrate content, charge, residual phenolics/protein and endotoxins. Chemical activity, receptor binding, uptake, digestion/fermentation, Nrf2 or inflammatory signaling and functional protection would then be compared using concentration–response modeling. Without this design, SAR remains a narrative association rather than a predictive relationship.

### 6.3. Acetylation and Sulfation Are Promising but Mechanistically Ambiguous

Acetylation is the strongest emerging modification line because two independent within-parent studies reported improved E1 activity, and the EMP→AEMP series extended the advantage to DSS colitis [26,28]. Sulfation likewise increases E1 activity and strengthened cellular protection in a PM_2.5_ model [45,50]. Both modifications alter several variables at once: electron distribution, ionic behavior, hydration, chain conformation, protein binding and microbial accessibility. Degree-of-substitution series, positional mapping and matched solubility controls are essential before a chemical group is called the active determinant. The current support and decisive experiments for the major candidate structure–activity determinants are summarized in Table 3.

## 7. Organ-Contextual Oxidative-Stress Protection

### 7.1. Nervous System and Neural Cells

PC12 and HEK293T studies consistently indicate preserved viability, lower ROS/MDA and less mitochondrial apoptosis after H_2_O_2_ challenge [10,29,31,43]. The 2026 APP/PS1 study adds a chronic neurodegenerative context in which a high-Mw MSP reduced amyloid burden and multiple regulated-cell-death programs [24]. The next step is not another PC12 viability study. Primary neurons, astrocytes and microglia should be tested in co-culture, with blood–brain exposure or gut–brain mediation measured explicitly. Redox protection should be separated from direct anti-amyloid or immunomodulatory effects.

### 7.2. Liver and Metabolic Disease

The liver has the broadest E4 evidence: D-galactose aging, CCl_4_ injury, chronic alcohol, NAFLD and more recent inflammatory hepatitis models [11,12,30,34,35,51]. Lower MDA/protein carbonyls and higher antioxidant enzymes often track improved enzymes, lipids, histology or fibrosis. Because the hepatic redox state is tightly coupled to substrate flux and immune infiltration, time-course experiments are critical. If lipid lowering precedes redox normalization, oxidative stress may be secondary; if Nrf2 or glutathione changes precede tissue protection and are required for it, a redox mechanism becomes more credible.

### 7.3. Intestine and Gut–Organ Axes

DSS and LPS studies show lower tissue MDA, higher SOD/CAT/GSH-Px or GSH and improved inflammatory/barrier outcomes, with acetylated material outperforming its parent in one modification series [28,36]. Other *Morchella* polysaccharides improve barrier and microbiota outcomes in colitis or diabetes without directly establishing redox mediation [47,52]. The intestine is a plausible primary site of action for large polymers; future work should measure the luminal and mucosal redox state, mucus penetration, epithelial Nrf2 requirement, fermentation products and microbiota causality in the same experiment.

### 7.4. Lung, Cardiac and Exercise Contexts

A549 and macrophage models show protection against H_2_O_2_ or PM_2.5_, suggesting relevance to epithelial and particulate injury [9,45]. NMCP-2 reduced doxorubicin-associated cardiac oxidative injury in cells and mice, but an interaction with doxorubicin exposure was not excluded [32]. In forced-swim mice, *Morchella* polysaccharides improved endurance-related outcomes with higher SOD/GSH-Px and lower MDA, alongside microbiome/metabolite shifts [37]. For exercise translation, redox biomarkers should be interpreted carefully because excessive antioxidant intervention can also blunt adaptive signaling; performance, recovery and training adaptation should be separated.

## 8. Critical Gaps and a Translational Roadmap

### 8.1. Identity, Purity and Reproducibility

The largest foundational weakness is structural heterogeneity. ‘Crude polysaccharide’, a named purified fraction and a chemically modified derivative are sometimes discussed as interchangeable doses. They are not. Every biological study should provide a material dossier: authenticated species and tissue/culture source; extraction and purification; carbohydrate, protein, phenolic, ash and nucleic-acid content; endotoxins; monosaccharide and linkage analysis; molecular-weight distribution; solution behavior; and batch acceptance criteria. Protein or phenolic depletion followed by activity reconstitution is particularly valuable when direct radical scavenging is claimed.

Endotoxin deserve explicit attention in macrophage, intestinal and inflammatory models. A nominally immunomodulatory polysaccharide can bind or carry lipopolysaccharide, and LPS challenge models are uniquely vulnerable to this confounding. Reporting a limulus or recombinant-factor-C result, recovery control and endotoxin units per administered dose should become routine.

### 8.2. Redox Measurement Must Be Orthogonal and Injury-Linked

Future studies should pair at least one direct oxidative-damage endpoint (e.g., lipid, protein or DNA oxidation) with a redox-buffer measure, an injury/functional endpoint and a mechanistic readout. MDA should not stand alone; F_2_-isoprostanes, protein carbonyls or 8-oxo-dG can provide orthogonal support depending on the model. ROS-sensitive dyes require probe-free controls and attention to polysaccharide fluorescence, adsorption and viscosity. Enzyme activities should be accompanied by expression or flux only when biologically justified.

### 8.3. Causal Mechanisms, Not Pathway Decoration

Most current mechanism claims are M0: a treatment improves the phenotype and changes p-Akt, Nrf2, HO-1, Bcl-2/BAX, NF-κB or NLRP3 in the expected direction. This is pathway concordance, not necessity. Priority experiments are Nrf2 loss-of-function for FMP-1-like materials; PI3K/Akt inhibition upstream; BAX/BAK or caspase interventions for mitochondrial claims; AMPK/Sirt1 perturbation in metabolic liver models; and inflammasome genetics for pyroptosis. A causal claim should require that pathway perturbation materially changes the polysaccharide effect and that nonspecific toxicity is excluded.

### 8.4. Exposure, Digestion and Microbiota

For oral products, the active entity may be intact polymer, absorbed oligomer, microbial metabolite or an altered microbial ecosystem. Size-exclusion and mass-spectrometric fingerprints should track the material through simulated digestion, fermentation, plasma and tissues where possible. Gut-mediator claims require depletion/transfer or defined-colonization experiments, not α-diversity alone. Dose should be normalized to the characterized carbohydrate and related to achievable food intake; parenteral cell exposure at milligram-per-milliliter concentrations should not be presented as evidence of oral bioavailability.

### 8.5. Preclinical Rigor and First-In-Human Design

Animal studies should prespecify primary outcomes, randomization, blinded assessment, sample-size rationale, exclusions, sex and batch and should follow ARRIVE 2.0 reporting [53]. A positive control can reveal whether the assay has a dynamic range; an inactive polysaccharide control can test structural specificity. Replication with an independently manufactured batch is more informative than adding another omics layer to one batch.

**Figure 4:**
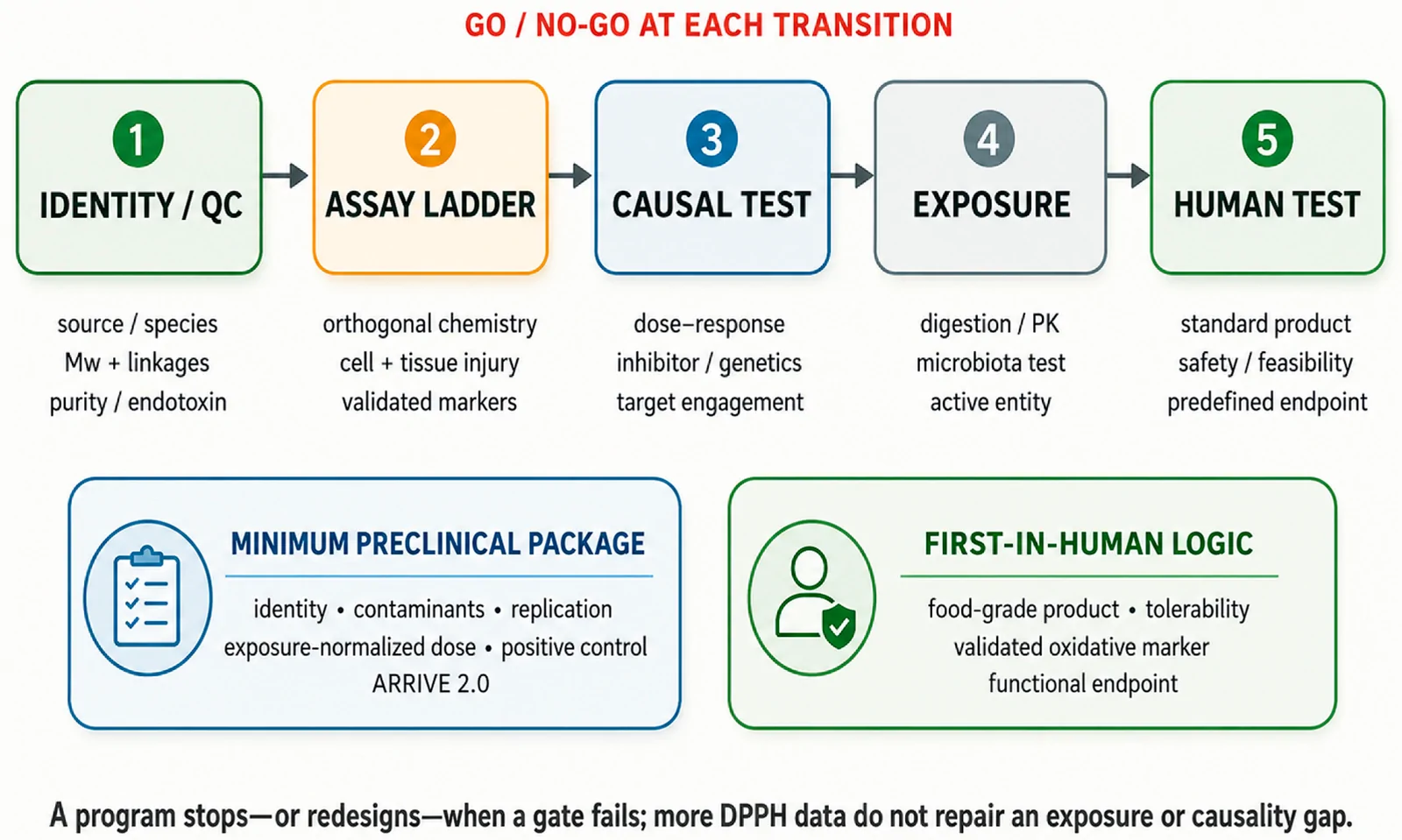
Translational roadmap with go/no-go gates. Progress depends on a reproducible material, assay orthogonality, the causal mechanism, plausible exposure and a standardized human product. Failure at a gate should trigger redesign rather than accumulation of additional chemical-radical assays.

A first human study should use a standardized, food-grade product with stability and safety data. A feasible initial design would be randomized, double-blind and placebo-controlled in a population with a defined but reversible oxidative burden. The primary endpoint should be one validated oxidative-damage marker chosen a priori, supported by exposure/fermentation markers and a context-specific functional measure. Multiple exploratory cytokines, microbiota features and antioxidant enzymes can be secondary, with false-discovery control. This design would test biological credibility without claiming disease treatment prematurely. These requirements are organized as sequential go/no-go gates in Figure 4.

## 9. Conclusions

The research question has matured from whether *Morchella* polysaccharides can reduce a laboratory radical to how structurally defined materials alter biological redox homeostasis. E1 chemistry is abundant; E3 and E4 studies increasingly demonstrate coordinated reductions in ROS or lipid and protein oxidation, restoration of endogenous defenses, mitochondrial preservation, and inflammatory resolution. PI3K/Akt–Nrf2/HO-1, AMPK/Sirt1, Bcl-2/BAX/caspase-3, NF-κB/NLRP3, and gut-mediated mechanisms form a set of recurrent and potentially interconnected pathways, but most reported links are associative rather than causally integrated.

The most defensible current conclusion is therefore conditional: selected *Morchella* polysaccharides and derivatives can protect experimental systems from oxidative injury, but the active structural determinant, proximal sensor, exposure route and pathway requirement are usually unresolved. Acetylation, controlled degradation and source-dependent composition offer promising SAR entry points. Decisive advances will come from identity-controlled materials; contaminant exclusion; within-parent structural series; causal perturbation; exposure and microbiota experiments; and, ultimately, E5 human trials. Until then, ‘redox-modulating biomacromolecule’ is a more accurate and productive description than ‘radical-scavenging antioxidant’.

## Figures and Tables

**Table 1:** Representative structure-resolved and structure-modified *Morchella* polysaccharides relevant to redox biology.

Identity	Source	Mw	Composition	Linkage/Conformation	Process	Redox Relevance
FMP-1	*M. esculenta*, fruiting body	4.7 kDa	Man:Glc:Gal ≈ 1:7.84:1.24	(1 → 4)-Glcp-rich backbone with (1 → 6)-Galp features	Native purified fraction	Defined low-Mw material used from chemical assays to zebrafish and A549 models [9,23]
NMCP-2	*M. conica*, fruiting body	48.3 kDa	Glucose-rich heteropolysaccharide	Structure-resolved neutral fraction; branched architecture reported	Native purified fraction	Links defined composition to mitochondrial protection in H_2_O_2_-challenged HEK293T cells [29]
MSP1-1	*M. sextelata*, fruiting body	389.0 kDa	Glucose-dominant	(1 → 4)-α-D-glucan with O-6 branching; random coil	Native purified fraction	Chemical antioxidant activity; useful comparator for size/conformation hypotheses [25].
MSP1-2	*M. sextelata*, fruiting body	23.4 kDa	Galactomannan plus α-glucan	Network-like conformation; structurally distinct from MSP1-1	Native purified fraction	Different architecture but the same study only supports E1 comparison [25].
MIP-D	*M. importuna*, fruiting body	2.6 MDa; 73 and 3.7 kDa populations	GlcN:Gal:Glc:Man = 0.39:1.88:3.82:3.91	Polydisperse; sulfate content of 34.16%	Deep-eutectic-solvent extraction	High yield and E1 activity, but multiple size populations complicate SAR attribution [20].
MSF	*M. esculenta*, fruiting body	234 kDa	Gal:Glc:Man = 4.34:90.22:5.45	Glucose-rich fraction	Native	Compared with fermentation-broth material in zebrafish dysbiosis/oxidative injury [22].
MSL	*M. esculenta*, fermentation broth	140 kDa	Ara:Gal:Glc:Man:GlcA = 0.31:14.71:13.03:71.43:0.53	Mannan-rich, trace uronic acid	Native	Fermentation-broth composition accompanied distinct redox and microbiota responses [22].
MEP2	*M. esculenta*, fruiting body	959 kDa	Glucose-rich	→4)-α-D-Glcp backbone with H-6 branching	Native purified fraction	Used in chronic alcohol injury; multi-omics implicated Usp10/NF-κB/Nrf2 [30].
MP/EMP/AEMP	*M. esculenta*, source material	19.6/5.67/6.35 kDa	Same major monosaccharide profile	Hydrolysis lowers Mw; acetylation changes substitution without changing monomer identity	Snailase degradation; acetylation	Stepwise modification increased E1 and DSS-colitis E4 effects; purity-matched head-to-head design is informative [28].
PMEP derivatives	*M. esculenta*, fruiting body	Not consistently exposure-matched	Parent composition retained	Sulfated, carboxymethylated or acetylated derivatives	Acetylation DS 0.40 ± 0.07	Acetylated derivative was strongest in E1 assays, but substitution also changes solubility and assay behavior [26].
Irradiated MSP	*Morchella* sp., fruiting body	Mw decreased with γ dose	Composition broadly retained	Chain scission and altered physical properties	Gamma irradiation	Enhanced E1 activity and H_2_O_2_-PC12 protection, supporting—without proving—a size effect [27].
MMP-L	*Morchella* mycelium	3.02 MDa	Gal:Glc:Man = 1:12.89:0.29	Complex α-linked glucose/galactose/mannose backbone and branches	Low-temperature extraction/purification	Large polymer protected H_2_O_2_-injured PC12 cells, arguing against a universal ‘smaller is better’ rule [10].
Mycelial MP	*Morchella* mycelium	5.7 kDa	96.555% glucose	Predominantly α-glycosidic glucan	Native	Alcoholic-liver model connected redox recovery with inflammasome/pyroptosis-associated transcripts [12].
MSP	*Morchella* sp.	2.283 MDa	Glucose-rich	→4)-α-Glcp and →4,6)-β-Glcp main-chain features with branches	Native purified fraction	APP/PS1 study linked a high-Mw structure to reduced PANoptosis; direct redox causality remains unresolved [24].

Abbreviations: Ara, arabinose; DS, degree of substitution; Gal, galactose; Glc, glucose; GlcA, glucuronic acid; GlcN, glucosamine; Man, mannose; Mw, molecular weight. Values are reported as described by the cited studies and should not be treated as directly comparable across analytical platforms.

**Table 2:** Representative biological evidence, mechanism score and major interpretive limitations. Studies are selected to span models and evidentiary strengths rather than to imply meta-analytic weighting.

Study	Grade	Structure/Material	Model/Inducer	Redox Outcomes	Mechanism Test	Interpretation	Main Limitation	Ref.
Huang 2012	E3/M1	EPMC/IPMC fractions	LPS–macrophage	NO/iNOS; HO-1; NF-κB	HO-1 inhibitor NaPP partially reversed NO/iNOS suppression; NF-κB effect persisted	HO-1 contributes but does not explain the full anti-inflammatory phenotype	50–200 μg/mL range; fraction identity and endotoxin controls require modern reporting	[14]
Fu 2013	E1 + E4/M0	MEEP; extracellular fraction	D-galactose-aged mice, 60 d	Serum/liver MDA; antioxidant enzymes; T-AOC	No pathway perturbation	Systemic redox association plus immune/aging endpoints	Crude/partly purified product; exposure and blinding not described in abstract	[34]
Xiong 2016	E3/M0	Crude MIP	H_2_O_2_–PC12	CAT, SOD, GSH-Px; MDA; caspase-3	Protein/phosphorylation associations only	NF-κB, p38/JNK ↓; ERK ↑	High model utility but no inhibitor/genetic confirmation	[31]
Cai 2018	E1 + E3/M0	FMP-1; 4.7 kDa, Glc-rich	AAPH–zebrafish embryos	ROS, NO, cell death, MDA; SOD/CAT/GSH-Px	No causal pathway test	Low-Mw defined fraction reduced oxidative injury in vivo like a simple organism	Embryo exposure bypasses mammalian absorption question	[23]
Li 2018	E3/M0	FMP-1; 4.7 kDa, Glc-rich	H_2_O_2_–A549	ROS/MDA; SOD/T-AOC; Cyt c/caspase-3	p-Akt, nuclear Nrf2 and HO-1 measured; no inhibitor/knockdown reported	PI3K/Akt–Nrf2/HO-1 is a supported hypothesis, not yet a demonstrated causal chain	Single cell line; no target engagement or uptake data	[9]
Xu 2018	E1 + E3/M0	NMCP-2; 48.3 kDa	H_2_O_2_–HEK293T	ROS; MMP; nuclear condensation	Bcl-2 ↑; BAX and cleaved caspase-3 ↓	Mitochondrial apoptosis modulation	No pathway perturbation; neutral fraction may act extracellularly	[29]
Li 2019	E3/M0	FMP-1 derivatives; sulfated SFMP-1	PM_2.5_–NR8383 macrophages	ROS; apoptosis; TNF-α/IL-1β; iNOS/COX-2	NF-κB nuclear translocation/IκB phosphorylation measured	Sulfation increased protection and anti-inflammatory activity	No matched cellular exposure, receptor or NF-κB perturbation	[45]
Xu 2021	E4/M0	MIP	CCl_4_–mouse liver	MDA, protein carbonyl; SOD/CAT; cytokines/MPO	Untargeted metabolomics; no perturbation	Redox and inflammatory recovery accompanied metabolic remodeling	Short 10 d exposure; female mice; no tissue distribution	[35]
Xu 2021	E3 + E4/M0	NMCP-2	Doxorubicin–H9C2 and mice	SOD/CAT/GSH; MDA	Bcl-2/BAX association	Cardiac redox protection and apoptosis attenuation	Anthracycline pharmacokinetic interaction not excluded	[32]
Teng 2023	E4/M0	MEP2; 959 kDa branched α-glucan	Chronic alcohol–mouse liver	Redox/inflammatory tissue endpoints	Proteome/metabolome/microbiome associations	Usp10/NF-κB/Nrf2 and gut–liver axis proposed	Multi-omics is hypothesis-generating unless mediator depletion/transfer is performed	[30]
Li 2024	E1 + E3/M0	MSF/MSL; *M. esculenta*; 234/140 kDa; Glc-/Man-rich	Metronidazole–zebrafish	SOD; Keap1/Nrf2 transcripts; microbiota	Transcript associations only	Fruiting-body and fermentation-broth preparations may produce different gut/redox profiles	No purified-feature matching; transcript changes are not Nrf2 activation	[22]
Zhang 2024	E4/M0	MEP	LPS–mouse intestine	MDA ↓; SOD/GSH-Px ↑; cytokines	Microbiota association; no depletion/transfer	Intestinal redox–inflammation crosstalk	LPS is an acute model; polymer purity and endotoxin controls are especially important	[36]
Chen 2025	E3/M0	MMP-L; 3.02 MDa, Glc-rich	H_2_O_2_–PC12	SOD/GSH; LDH/MDA/ROS; apoptosis	PI3K/Akt and Bcl-2/BAX/caspase-3 associations	Large mycelial polymer protected neural cells	Dose of 0.05–3.2 mg/mL; inhibitor/knockdown and osmolarity controls needed	[10]
Ma 2025	E1 + E4/M0	MP → EMP/AEMP; 19.6 → 5.7/6.4 kDa	DSS–mouse colitis	SOD/CAT/GSH-Px; MDA; cytokines	Modification series; no pathway perturbation	Hydrolysis plus acetylation improved redox and colitis outcomes	Best available within-study SAR logic, but degree-of-substitution and exposure effects remain coupled	[28]
Wang 2025	E4/M0	*Morchella* polysaccharide	HFD and MCD NAFLD mice	Lipid, inflammation and fibrosis outcomes	AMPK/Sirt1 abundance/activity association	Metabolic protection may restore redox homeostasis indirectly	Direct oxidative-damage endpoints and pathway inhibition are needed	[11]
Hu 2026	E4/M0	Mycelial MP; 5.7 kDa, 96.6% Glc	Alcoholic-liver mouse	SOD/CAT/GSH ↑; MDA ↓	Transcriptomic NLRP3/Ifi16/Pycard/pro-caspase-1 associations	Redox recovery coupled to reduced pyroptosis/inflammation	No NLRP3 inhibitor/KO, exposure or microbiota test	[12]
Liu 2026	E4/M0	Crude MP/MP1-1; (1 → 4)-α-glucan	Forced-swim mouse, 4 weeks	SOD/GSH-Px ↑; MDA ↓; inflammatory markers	Microbiome/metabolite correlations	Anti-fatigue performance accompanied by redox and gut changes	Crude product used in vivo; no antibiotic depletion/FMT; 50–200 mg/kg	[37]
Gao 2026	E3/M0	Cap vs. stipe polysaccharides	H_2_O_2_–*C. elegans*	Survival, ROS/MDA; gst-4/sod-3/gsh-px	Gene-expression association	Simple-organism bridge between E1 and mammalian studies	RNAi/knockout and bacterial-diet controls would strengthen attribution	[33]
Su 2026 †	E4/M2	MCP	LAD–mouse myocardium	Inflammation and remodeling; 12-HEPE	Antibiotic cocktail abolished efficacy	Causal gut-microbiota dependence demonstrated	Mechanistically adjacent rather than a direct redox study; illustrates needed causal design	[15]

† Mechanistically adjacent study retained to illustrate causal gut-mediator testing. Grades: E1, chemical assay; E3, cell/simple organism; E4, mammalian model; M0, association; M1, pharmacological perturbation; M2, genetic/transfer/depletion or direct target engagement. AAPH, 2,2′-azobis(2-amidinopropane) dihydrochloride; CCl_4_, carbon tetrachloride; Cyt c, cytochrome c; DSS, dextran sulfate sodium; HFD, high-fat diet; LAD, left anterior descending coronary artery; MCD, methionine/choline-deficient diet; MMP, mitochondrial membrane potential; MPO, myeloperoxidase; T-AOC, total antioxidant capacity.

**Table 3:** Candidate structure–activity determinants, present evidence and decisive experiments.

Determinant	Plausible Mechanism	Current Evidence	Confidence	Decisive Experiment
Molecular weight	Lower Mw may improve solubility, diffusibility and reactive-end accessibility; high Mw may strengthen receptor clustering or gut retention.	Irradiation, enzymatic and subcritical-water preparations often show stronger E1/E3 effects; MMP-L and MSP remain active at multi-MDa scale [10,21,24,27]	Moderate; non-monotonic	Prepare a narrow Mw series from one parent polymer; match substitution, charge, purity and molar/monomer dose; measure uptake and receptor binding.
Monosaccharide composition	Glucose-rich α-glucans may engage pattern-recognition receptors; Gal/Man/uronic-acid content changes hydration, charge and fermentation.	*M. esculenta* MSF/MSL and cap/stipe comparisons associate different compositions with different redox phenotypes [22,33]	Low	Use purified, linkage-defined fractions and receptor panels; avoid composition-only correlations across species/extractions.
Glycosidic linkage and branching	Branch density changes chain flexibility, epitope valency, viscosity and enzyme accessibility.	FMP-1, MSP1-1/2, MEP2 and recent high-Mw fractions provide structural contrasts, but few are compared on one assay platform [23,25,30].	Low–moderate	Generate controlled debranching or linkage-selective fragments; confirm by methylation/NMR and test signaling at equal free-sugar equivalents.
Conformation/aggregation	Triple-helical, coil and network states may alter radical accessibility and receptor avidity; aggregation can distort nominal concentration.	MSP1-1 random coil and MSP1-2 network illustrate conformational diversity, but only E1 activity was measured [25].	Low	Characterize in the actual assay medium by SEC-MALS, DLS, Congo-red or scattering; correlate monomeric/aggregate state with activity.
Uronic acid/sulfate	Anionic groups can enhance water solubility, chelation and electrostatic interactions.	Native sulfated MIP-D and sulfated derivatives show strong E1 activity; sulfation also enhanced PM_2.5_-cell protection [20,45,50].	Moderate for E1; low for biology	Use matched degree-of-substitution series, ionic-strength controls and sulfate-only controls; quantify cellular exposure and endotoxins.
Acetylation	Acetyl groups may change electron density, hydrophobicity, conformation, membrane association and fermentability.	Acetylated PMEP and AEMP outperformed parent materials in E1 assays; AEMP was strongest in DSS mice [26,28].	Moderate	Map substitution position/degree, solubility and PK; compare acetylated and deacetylated rescue materials in the same disease model.
Extraction/degradation	Process severity changes Mw, branching, protein/phenolic carryover and reducing-end abundance simultaneously.	Enzyme-assisted, deep-eutectic and subcritical-water methods increase yield/activity, but structural and contaminant changes are entangled [19,20,21].	Moderate for process–activity; low for a single determinant	Mass-balance every fraction; quantify phenolics/protein/endotoxin; use orthogonal purification and reconstitution controls.
Residual small molecules	Phenolics, peptides, Maillard products or salts can dominate E1 signals and create false SAR.	Many reports provide carbohydrate content but not exhaustive small-molecule or endotoxin exclusion; crude and purified materials are sometimes discussed together.	High plausibility; under-tested	Dialysis/ultrafiltration, polyphenol/protein assays, LC–MS fingerprinting, endotoxin testing and activity-guided depletion/reconstitution.

## Data Availability

No new data were created or analyzed in this study. Data sharing is not applicable to this article.

## Abbreviations

AAPH, 2,2′-azobis(2-amidinopropane) dihydrochloride; ABTS, 2,2′-azino-bis(3-ethylbenzothiazoline-6-sulfonic acid); AMPK, AMP-activated protein kinase; CAT, catalase; DPPH, 2,2-diphenyl-1-picrylhydrazyl; DSS, dextran sulfate sodium; GSH, glutathione; GPx/GSH-Px, glutathione peroxidase; HO-1, heme oxygenase-1; MDA, malondialdehyde; MMP, mitochondrial membrane potential (where used as an endpoint); Mw, molecular weight; NF-κB, nuclear factor kappa B; NQO1, NAD(P)H quinone dehydrogenase 1; Nrf2, nuclear factor erythroid 2-related factor 2; PK, pharmacokinetics; ROS, reactive oxygen species; SAR, structure–activity relationship; Sirt1, sirtuin 1; SOD, superoxide dismutase; T-AOC, total antioxidant capacity.
